# 1,3-Bis(2,4,6-trimethyl­phen­yl)-3*H*-imidazol-1-ium tetra­oxidorhenate(VII)

**DOI:** 10.1107/S1600536811050677

**Published:** 2011-11-30

**Authors:** Marilé Landman, Belinda van der Westhuizen, Daniela I. Bezuidenhout, David C. Liles

**Affiliations:** a Department of Chemistry, University of Pretoria, Private Bag X20, Hatfield 0028, South Africa

## Abstract

The title compound, (C_21_H_25_N_2_)[ReO_4_], was formed as the unexpected product in an attempted synthesis of a rhenium(I)–*N*-heterocyclic carbene (NHC) complex. The compound has crystallographic mirror symmetry with both the cation and the tetrahedral anion located across a mirror plane. The cation and anion are linked by a C—H⋯O hydrogen bond.

## Related literature

For related structures of some halide salts, see: Arduengo *et al.* (1995 ▶); Cole *et al.* (2002 ▶); Cole & Junk (2004 ▶); Lorber & Vendier (2009 ▶).
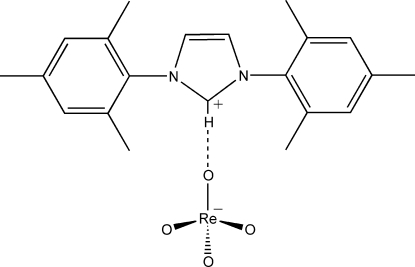


## Experimental

### 

#### Crystal data

(C_21_H_25_N_2_)[ReO_4_]
*M* *_r_* = 555.63
Monoclinic, 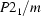

*a* = 8.2989 (12) Å
*b* = 16.373 (2) Å
*c* = 8.3168 (12) Å
β = 111.948 (2)°
*V* = 1048.2 (3) Å^3^
*Z* = 2
Mo *K*α radiation
μ = 5.83 mm^−1^
*T* = 293 K
0.39 × 0.10 × 0.09 mm

#### Data collection

Bruker (Siemens) P4 diffractometer with a Bruker SMART 1000 CCD detector
Absorption correction: multi-scan (*SADABS*; Bruker, 2001 ▶) *T*_min_ = 0.489, *T*_max_ = 0.592
5688 measured reflections
2056 independent reflections
1895 reflections with *I* > 2σ(*I*)
*R*_int_ = 0.029

#### Refinement

*R*[*F* ^2^ > 2σ(*F* ^2^)] = 0.033
*wR*(*F* ^2^) = 0.083
*S* = 1.13
2056 reflections
136 parameters
H-atom parameters constrained
Δρ_max_ = 1.32 e Å^−3^
Δρ_min_ = −0.86 e Å^−3^

### 

Data collection: *SMART* (Bruker, 2001 ▶); cell refinement: *SAINT* (Bruker, 2001 ▶); data reduction: *SAINT*; program(s) used to solve structure: *SHELXTL* (Sheldrick, 2008 ▶); program(s) used to refine structure: *SHELXTL*; molecular graphics: *ORTEP-3 for Windows* (Farrugia, 1997 ▶) and *POV-RAY* (Cason, 2004 ▶); software used to prepare material for publication: *SHELXTL* and *PLATON* (Spek, 2009 ▶).

## Supplementary Material

Crystal structure: contains datablock(s) I, global. DOI: 10.1107/S1600536811050677/ez2273sup1.cif

Structure factors: contains datablock(s) I. DOI: 10.1107/S1600536811050677/ez2273Isup2.hkl

Supplementary material file. DOI: 10.1107/S1600536811050677/ez2273Isup3.cdx

Additional supplementary materials:  crystallographic information; 3D view; checkCIF report

## Figures and Tables

**Table 1:** Hydrogen-bond geometry (Å, °)

*D*—H⋯*A*	*D*—H	H⋯*A*	*D*⋯*A*	*D*—H⋯*A*
C1—H1⋯O2^i^	0.93	2.21	3.12 (1)	167 (1)

Symmetry code: (i) 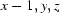.

**Fig. 1.**
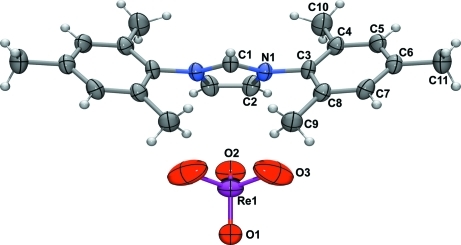
The molecular structure of 1 showing the atomic numbering scheme. Displacement elipsoids are shown at the 50% probability level and H atoms are shown as small spheres of arbitary radii.

## supplementary crystallographic information

### Comment

1,3-Bis(2,4,6-trimethylphenyl)-3*H*-imidazolium tetraoxorhenate(VII) (IMesH[ReO_4_], 1) was formed as the unexpected product in an attempted synthesis of a rhenium(I)-*N*-heterocyclic carbene (NHC) complex. The reaction pathway is unexplained. The main feature in the formation of this complex is the oxidation of the rhenium metal from the +1 to the +7 state resulting in a [ReO_4_]^-^ anion. This anion replaces the Cl^-^ anion in the IMesHCl salt since it is larger and the co-crystallization of more similar sized ions is favoured..

The compound has crystallographic mirror symmetry with the Re1, O1, O2 and the imidazolium C1 and H1 atoms all lying in a mirror plane. The geometry of the imidazolium cation is similar to those observed for previously reported structures of 1,3-Bis(2,4,6-trimethylphenyl)-3*H*-imidazolium salts, for example halide (Cl^-^, Br^-^) salts (Arduengo *et al.*, 1995; Cole *et al.*, 2002; Cole & Junk, 2004; Lorber &Vendier, 2009). The imidazolium C1—H1 is hydrogen bonded to O2 of the [ReO_4_]^-^ anion (at *x* - 1, *y*, *z*) with H1···O2 = 2.21 (1) Å and C1—H1···O2 = 167 (1)°. Similar hydrogen bonds were observed between the cation and the anion in the halide salts (Cole & Junk, 2004). The Re—O2 bond is somewhat longer (1.714 (5) Å) than the other (nominally double) Re—O bonds (mean 1.690 (6) Å) and may indicate some localization of the anion negative charge on O2.

### Experimental

*N*,*N*'-Dimesitylimidazolium chloride (IMesHCl, 2 mmol, 0.66 g) was deprotonated with KO*^t^*Bu in tetrahydrofuran (thf) solution. This mixture was added to a thf solution of [Re_2_(CO)_10_] (1.06 mmol, 0.68 g) and stirred. Thin layer chromatography (TLC) was used to monitor the reaction and, due to the initial lack of product formation, the reaction mixture was heated in an oilbath at 70°C for an hour. The thf solvent was removed leaving a brown residue. The product was extracted with hexane and the hexane was then removed under reduced pressure. The residue was purified by column chromatography. A dark yellow fraction was eluted with dichloromethane (dcm). Crystallization from a 1:1 dcm:hexane solution gave light brown crystals of IMesH[ReO_4_] (1). 1H NMR (δ, p.p.m.), C_6_D_6_: 1.91 (br,12*H*), 2.19 (br, 6H), 6.70 (br, 4H), 7.14 (s, 2H); 13 C NMR (δ, p.p.m.), C_6_D_6_: 17.8, 20.9, 121.8, 126.9, 128.9, 129.6, 141.2

### Refinement

All hydrogen atoms were added in calculated positions. Each was allowed to ride on the atom to which it is bonded with an isotropic adp set to 1.2 *x* (1.5 *x* for methyl H atoms) the equivalent isotropic adp of that atom.

### Crystal data

**Table:**

(C_21_H_25_N_2_)[ReO_4_]	*F*(000) = 544
*M**_r_* = 555.63	*D*_x_ = 1.760 Mg m^−^^3^
Monoclinic, *P*2_1_/*m*	Mo *K*α radiation, λ = 0.71073 Å
Hall symbol: -P 2yb	Cell parameters from 4658 reflections
*a* = 8.2989 (12) Å	θ = 2.6–26.5°
*b* = 16.373 (2) Å	µ = 5.83 mm^−^^1^
*c* = 8.3168 (12) Å	*T* = 293 K
β = 111.948 (2)°	Needle, light brown
*V* = 1048.2 (3) Å^3^	0.39 × 0.10 × 0.09 mm
*Z* = 2	

### Data collection

**Table:**

Bruker (Siemens) P4 diffractometer	2056 independent reflections
Radiation source: fine-focus sealed tube	1895 reflections with *I* > 2σ(*I*)
graphite	*R*_int_ = 0.029
Detector resolution: 8.3 pixels mm^-1^	θ_max_ = 26.5°, θ_min_ = 2.5°
φ and ω scans	*h* = −10→9
Absorption correction: multi-scan (*SADABS*; Bruker, 2001)	*k* = −19→20
*T*_min_ = 0.489, *T*_max_ = 0.592	*l* = −3→10
5688 measured reflections	

### Refinement

**Table:**

Refinement on *F*^2^	Primary atom site location: structure-invariant direct methods
Least-squares matrix: full	Secondary atom site location: difference Fourier map
*R*[*F*^2^ > 2σ(*F*^2^)] = 0.033	Hydrogen site location: inferred from neighbouring sites
*wR*(*F*^2^) = 0.083	H-atom parameters constrained
*S* = 1.13	*w* = 1/[σ^2^(*F*_o_^2^) + (0.0381*P*)^2^ + 1.6122*P*] where *P* = (*F*_o_^2^ + 2*F*_c_^2^)/3
2056 reflections	(Δ/σ)_max_ < 0.001
136 parameters	Δρ_max_ = 1.32 e Å^−^^3^
0 restraints	Δρ_min_ = −0.86 e Å^−^^3^

### Special details

**Table:**

Geometry. All e.s.d.'s (except the e.s.d. in the dihedral angle between two l.s. planes) are estimated using the full covariance matrix. The cell e.s.d.'s are taken into account individually in the estimation of e.s.d.'s in distances, angles and torsion angles; correlations between e.s.d.'s in cell parameters are only used when they are defined by crystal symmetry. An approximate (isotropic) treatment of cell e.s.d.'s is used for estimating e.s.d.'s involving l.s. planes.
Refinement. Refinement of *F*^2^ against ALL reflections. The weighted *R*-factor *wR* and goodness of fit *S* are based on *F*^2^, conventional *R*-factors *R* are based on *F*, with *F* set to zero for negative *F*^2^. The threshold expression of *F*^2^ > σ(*F*^2^) is used only for calculating *R*-factors(gt) *etc*. and is not relevant to the choice of reflections for refinement. *R*-factors based on *F*^2^ are statistically about twice as large as those based on *F*, and *R*- factors based on ALL data will be even larger.All hydrogen atoms were added in calculated positions. Each was allowed to ride on the atom to which it is bonded with an isotropic adp set to 1.2 *x* (1.5 *x* for methyl H atoms) the equivalent isotropic adp of that atom.

### Fractional atomic coordinates and isotropic or equivalent isotropic displacement parameters (Å^2^)

**Table:**

	*x*	*y*	*z*	*U*_iso_*/*U*_eq_	
N1	0.3511 (4)	0.3161 (2)	0.0558 (4)	0.0350 (7)	
C1	0.2687 (8)	0.2500	0.0779 (7)	0.0332 (11)	
H1	0.1694	0.2500	0.1046	0.040*	
C2	0.4899 (6)	0.2909 (3)	0.0166 (6)	0.0441 (10)	
H2	0.5694	0.3245	−0.0058	0.053*	
C3	0.3080 (5)	0.3994 (2)	0.0842 (5)	0.0340 (8)	
C4	0.2575 (6)	0.4545 (2)	−0.0539 (5)	0.0392 (9)	
C5	0.2133 (6)	0.5328 (3)	−0.0207 (6)	0.0425 (9)	
H5	0.1779	0.5705	−0.1109	0.051*	
C6	0.2198 (6)	0.5569 (2)	0.1404 (6)	0.0402 (9)	
C7	0.2735 (6)	0.5004 (2)	0.2743 (6)	0.0408 (9)	
H7	0.2783	0.5160	0.3835	0.049*	
C8	0.3202 (6)	0.4215 (2)	0.2506 (5)	0.0370 (9)	
C9	0.3843 (7)	0.3635 (3)	0.4026 (6)	0.0476 (11)	
H9A	0.4806	0.3326	0.3976	0.071*	
H9B	0.2923	0.3270	0.3982	0.071*	
H9C	0.4209	0.3941	0.5088	0.071*	
C10	0.2518 (9)	0.4325 (3)	−0.2318 (6)	0.0603 (14)	
H10A	0.1737	0.4687	−0.3159	0.091*	
H10B	0.2119	0.3772	−0.2584	0.091*	
H10C	0.3660	0.4375	−0.2345	0.091*	
C11	0.1718 (8)	0.6422 (3)	0.1711 (8)	0.0561 (13)	
H11A	0.0786	0.6404	0.2131	0.084*	
H11B	0.1352	0.6724	0.0644	0.084*	
H11C	0.2707	0.6686	0.2555	0.084*	
Re1	0.91490 (4)	0.2500	0.40489 (3)	0.05388 (13)	
O1	1.0911 (8)	0.2500	0.5917 (7)	0.0756 (18)	
O2	0.9768 (8)	0.2500	0.2300 (8)	0.0769 (17)	
O3	0.7942 (8)	0.3344 (5)	0.3947 (7)	0.127 (2)	

### Atomic displacement parameters (Å^2^)

**Table:**

	*U*^11^	*U*^22^	*U*^33^	*U*^12^	*U*^13^	*U*^23^
N1	0.0437 (19)	0.0286 (16)	0.0382 (17)	−0.0031 (14)	0.0216 (15)	−0.0005 (13)
C1	0.038 (3)	0.026 (3)	0.041 (3)	0.000	0.021 (2)	0.000
C2	0.050 (2)	0.039 (2)	0.056 (3)	−0.009 (2)	0.034 (2)	−0.002 (2)
C3	0.043 (2)	0.0231 (17)	0.042 (2)	−0.0053 (16)	0.0221 (18)	−0.0030 (15)
C4	0.047 (2)	0.032 (2)	0.041 (2)	−0.0073 (18)	0.0204 (19)	−0.0007 (17)
C5	0.051 (3)	0.031 (2)	0.045 (2)	−0.0054 (18)	0.017 (2)	0.0048 (17)
C6	0.043 (2)	0.029 (2)	0.051 (2)	−0.0043 (17)	0.0203 (19)	−0.0030 (17)
C7	0.051 (3)	0.035 (2)	0.042 (2)	−0.0067 (18)	0.024 (2)	−0.0105 (17)
C8	0.042 (2)	0.033 (2)	0.040 (2)	−0.0068 (17)	0.0191 (18)	−0.0027 (16)
C9	0.062 (3)	0.044 (2)	0.039 (2)	−0.003 (2)	0.021 (2)	0.0002 (18)
C10	0.096 (4)	0.049 (3)	0.043 (2)	−0.006 (3)	0.035 (3)	−0.002 (2)
C11	0.071 (3)	0.035 (2)	0.068 (3)	0.003 (2)	0.032 (3)	−0.005 (2)
Re1	0.04629 (19)	0.0774 (2)	0.04186 (17)	0.000	0.02096 (13)	0.000
O1	0.077 (4)	0.060 (3)	0.072 (4)	0.000	0.008 (3)	0.000
O2	0.077 (4)	0.107 (5)	0.060 (3)	0.000	0.041 (3)	0.000
O3	0.116 (5)	0.179 (6)	0.081 (3)	0.073 (5)	0.032 (3)	0.004 (4)

### Geometric parameters (Å, °)

**Table:**

N1—C1	1.330 (5)	C7—H7	0.9300
N1—C2	1.372 (5)	C8—C9	1.510 (6)
N1—C3	1.452 (5)	C9—H9A	0.9600
C1—N1^i^	1.330 (5)	C9—H9B	0.9600
C1—H1	0.9300	C9—H9C	0.9600
C2—C2^i^	1.341 (9)	C10—H10A	0.9600
C2—H2	0.9300	C10—H10B	0.9600
C3—C4	1.395 (6)	C10—H10C	0.9600
C3—C8	1.397 (6)	C11—H11A	0.9600
C4—C5	1.390 (6)	C11—H11B	0.9600
C4—C10	1.506 (6)	C11—H11C	0.9600
C5—C6	1.379 (6)	Re1—O1	1.689 (5)
C5—H5	0.9300	Re1—O3	1.691 (6)
C6—C7	1.388 (6)	Re1—O3^i^	1.691 (6)
C6—C11	1.500 (6)	Re1—O2	1.714 (5)
C7—C8	1.385 (6)		
			
C1—N1—C2	108.0 (4)	C3—C8—C9	122.7 (4)
C1—N1—C3	124.8 (3)	C8—C9—H9A	109.5
C2—N1—C3	126.9 (3)	C8—C9—H9B	109.5
N1—C1—N1^i^	109.0 (5)	H9A—C9—H9B	109.5
N1—C1—H1	125.5	C8—C9—H9C	109.5
N1^i^—C1—H1	125.5	H9A—C9—H9C	109.5
C2^i^—C2—N1	107.5 (2)	H9B—C9—H9C	109.5
C2^i^—C2—H2	126.3	C4—C10—H10A	109.5
N1—C2—H2	126.3	C4—C10—H10B	109.5
C4—C3—C8	122.4 (4)	H10A—C10—H10B	109.5
C4—C3—N1	119.3 (3)	C4—C10—H10C	109.5
C8—C3—N1	118.2 (3)	H10A—C10—H10C	109.5
C5—C4—C3	117.0 (4)	H10B—C10—H10C	109.5
C5—C4—C10	120.1 (4)	C6—C11—H11A	109.5
C3—C4—C10	122.9 (4)	C6—C11—H11B	109.5
C6—C5—C4	122.7 (4)	H11A—C11—H11B	109.5
C6—C5—H5	118.7	C6—C11—H11C	109.5
C4—C5—H5	118.7	H11A—C11—H11C	109.5
C5—C6—C7	118.2 (4)	H11B—C11—H11C	109.5
C5—C6—C11	121.1 (4)	O1—Re1—O3	109.8 (2)
C7—C6—C11	120.7 (4)	O1—Re1—O3^i^	109.8 (2)
C8—C7—C6	122.1 (4)	O3—Re1—O3^i^	109.7 (6)
C8—C7—H7	118.9	O1—Re1—O2	110.5 (3)
C6—C7—H7	118.9	O3—Re1—O2	108.5 (2)
C7—C8—C3	117.5 (4)	O3^i^—Re1—O2	108.5 (2)
C7—C8—C9	119.8 (4)		
			
C2—N1—C1—N1^i^	0.6 (6)	C3—C4—C5—C6	0.7 (7)
C3—N1—C1—N1^i^	−174.5 (3)	C10—C4—C5—C6	−178.5 (5)
C1—N1—C2—C2^i^	−0.3 (4)	C4—C5—C6—C7	0.3 (7)
C3—N1—C2—C2^i^	174.6 (3)	C4—C5—C6—C11	179.7 (4)
C1—N1—C3—C4	−119.1 (5)	C5—C6—C7—C8	0.0 (7)
C2—N1—C3—C4	66.8 (6)	C11—C6—C7—C8	−179.4 (4)
C1—N1—C3—C8	61.3 (6)	C6—C7—C8—C3	−1.4 (6)
C2—N1—C3—C8	−112.7 (5)	C6—C7—C8—C9	177.6 (4)
C8—C3—C4—C5	−2.1 (6)	C4—C3—C8—C7	2.4 (6)
N1—C3—C4—C5	178.3 (4)	N1—C3—C8—C7	−178.0 (4)
C8—C3—C4—C10	177.1 (5)	C4—C3—C8—C9	−176.5 (4)
N1—C3—C4—C10	−2.4 (6)	N1—C3—C8—C9	3.0 (6)

Symmetry codes: (i) *x*, −*y*+1/2, *z*.

### Hydrogen-bond geometry (Å, °)

**Table:**

*D*—H···*A*	*D*—H	H···*A*	*D*···*A*	*D*—H···*A*
C1—H1···O2^ii^	0.93	2.21	3.12 (1)	167.(1)

Symmetry codes: (ii) *x*−1, *y*, *z*.
